# A comparison of risk factors and severity of ischemic stroke in female and male genders in North-West Iran: A cross-sectional study

**Published:** 2014-10-06

**Authors:** Mahnaz Talebi, Mohammad Ghertasi, Aliakbar Taheraghdam, Sasan Andalib, Ehsan Sharifipour

**Affiliations:** Department of Neurology, Neurosciences Research Center (NSRC), School of Medicine, Tabriz University of Medical Sciences, Tabriz, Iran

**Keywords:** Ischemic Stroke, Gender, Risk Factor

## Abstract

**Background: **Gender difference has been reported in stroke risk factors and disease history. The aim of this study was to compare risk factors and the severity of ischemic stroke based upon modified Rankin Scale (mRS) and hospital mortality between two genders.

**Methods:** In a cross-sectional study, 341 patients (44% males and 56% females with a mean age of 68.94 ± 12.74 years) with ischemic stroke, who were hospitalized in the neurology wards of two referral university hospital of North-West Iran (Imam Reza and Razi Hospitals), from the beginning to the end of 2011 were selected and assessed. Gender difference in terms of demographic findings, vascular risk factors, 7^th^ day mRS, and hospital mortality (during admission) were evaluated.

**Results: **In 2.6% of cases, mRS was found to be less than 2 (favorable) and in 97.4% of cases; mRS was 2-5 (with disability). No significant difference in ischemic stroke severity based on mRS was observed between two genders. There was a significant difference in the rate of hypertension (females = 72.3%, males = 59.3%, P = 0.010), diabetes (females = 28.8%, males = 18.7%, P = 0.030), smoking (females = 6.3%, males = 35.3%, P < 0.001). No significant difference was seen in other risk factors between two genders. There was no significant difference in the mortality rate, which constituted 8.9% and 4.7% in females and males respectively (P = 0.140).

**Conclusion: **The evidence from the present study suggests that despite the existence of some difference between risk-factors in two genders, there was no difference in terms of ischemic stroke severity and mortality rate between two genders.

## Introduction

Stroke, which is mostly seen in the old, has been one of the most crucial discussion along with an increase in the age of human societies. Stroke is one of the leading causes of mortality after the age of 55 years, incidence doubles every decade.^1^^-^^3^ Several factors involved in stroke prognosis.^4^ The evidence from several studies indicates that females experience unfavorable outcome subsequent to stroke, compared with males. They also suffer stroke-related death in older groups.^5^^-^^7^ Furthermore, previous studies from Iran reported higher hospital mortality rate in Iran.^8^

The probability of discharge from hospital is less in females, and they show increased activity limitations and functional damage during follow-up.^9^^-^^11^ Following a stroke, females may experience psychological problems, depression, fatigue, and a low life quality, in comparison with males.^12^^-^^14^ Cohort studies demonstrate gender difference in stroke symptoms and history;^2^^,^^5^^,^^15^^,^^16^ for instance, females experience higher rate of atrial fibrillation (AF) and hypertension;^9^^,^^10^^,^^17^ even so, males mostly suffer diabetes^9^ and cardiac disease excepting AF.^4^ With regard to racial differences, hypertension was the most common risk factor for ischemic stroke in reports from Iran (Tehran and Khorasan).^18^^,^^19^ Our aim was to assess risk factors’ difference, the severity of ischemic stroke based upon modified Rankin Scale (mRS) criteria,^17^ and in-hospital mortality in both genders.

## Materials and Methods

In a cross-sectional study, 341 patients with ischemic stroke, who were referred and hospitalized in the neurology wards of two referral university hospital of North-West Iran (Imam Reza and Razi Hospitals), Tabriz University of Medical Sciences from the beginning to the end of 2011 were selected and assessed. All patients hospitalized due to ischemic stroke over the 1 year were included in the study; however, patients with subarachnoid and intra-parenchymal hemorrhages, subdural or extradural hemorrhages, and traumatic or neoplastic damages and those who had been received thrombolytic therapy were excluded from the study. All the patients were assessed by two neurologists, and the results were recorded in the questionnaires. Stroke risk factors, including age, gender, family history, diabetes, smoking, drinking, hypertension, hyperlipidemia, cardiac diseases, oral contraceptive pills (OCP) consumption, and collagen vascular disease were assessed by using the questionnaires. The severity of ischemic stroke based on mRS was also recorded in each patient in the 7^th^ day of admission, as well as the hospital mortality rate (during the first admission after acute ischemic stroke). In the mRS of lower than 2, the patients failed to have disability, whereas, in the mRS of between 2 and 5, the patients were disable as a consequence of stroke.

Data were analyzed using SPSS for Windows 16.0 (SPSS Inc., Chicago, IL, USA) Mean, percentage, and mean ± standard deviation were provided as descriptive statistics. In order to compare two genders, chi-square or Fisher exact tests were used for qualitative variables; even so, independent t-test was used for quantitative variables. Moreover, logistic regression analysis was used for predictive factors, and a P-value of less than 0.05 was considered to be statistical significance.

## Results

**Figure 1:**
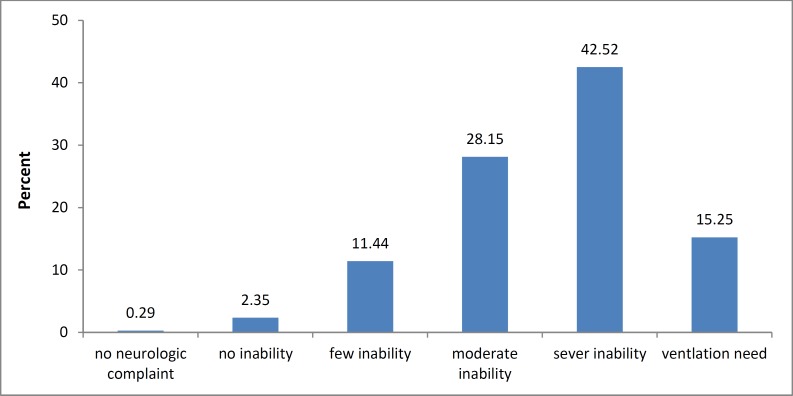
The frequency of modified Rankin Scale in the stroke patients

Of the 341 patients with ischemic stroke, there were 150 male (44%) and 191 female (56%). The mean age of the patients was 68.94 ± 12.74 years (27-97). No significant difference in the mean age of male and female patients, family history of stroke, history of stroke, hyperlipidemia, AF and other cardiac disease and drinking was observed between two genders (Table 1). Previous transient ischemic attack (TIA) history existed in 5 cases (1.5%). 3 (2%) in male and 2 (1%) in female. Hypertension was seen in 227 patients (66.6%). The mean duration of hypertension was 10.42 ± 6.52 years. Female had hypertension more than male (P = 0.010). There was no significant difference in previous cardiac disease history between male and female (P = 0.730) (Table 1). A total of 65 stroke patients (19.1%) were smokers with a mean duration of 31.12 ± 15.62 years. Smoking rate was very higher in male (53 cases) than in female (12 cases) (P < 0.001). Diabetes was found in 83 stroke patients (24.3%), and was observed to be more prevalent in female (55 cases) than in male (28 cases) P = 0.030. OCP was consumed in 10 female (5.2%) with a mean duration of 2.50 ± 2.12 years. Collagen vascular diseases existed in 8 stroke patients (2.3%), including 2 female (1.3%) and 6 male (3.1%). A mean mRS of 3.56 ± 3.56 with a mode of 4 was found in the stroke patients. Figure 1 illustrates the frequency of mRS in the stroke patients. As can be noted, the majority of stroke patients experienced moderately to severe disability. Nine stroke patients (2.6%) showed mRS of < 2 (favorable mRS), 332 cases (97.4%) with mRS of 2-5 (stroke patients with disability). The mean mRS in male and female was 3.46 ± 1.01 and 3.63 ± 0.94, respectively (P = 0.090). 

**Table 1:** The comparison of ischemic stroke risk factors according to sex

**Risk factors**	**Men, n = 150 (44%)**	**Women, n = 191 (56%)**	**P**
Age (years)	67.69 ± 12.69*	69.92 ± 12.72*	0.110
Positive family history of stroke, n (%)	12 (8.0)	17 (8.90)	0.840
Previous stroke or TIA, n (%)	41 (27.3)	41 (21.40)	0.290
Hypertension, n (%)	39 (26.1)	77 (40.47)	0.010
Hyperlipidemia, n (%)	24 (16.0)	39 (20.40)	0.320
Ischemic heart disease, n (%)	23 (15.3)	29 (15.10)	0.700
Volvular heart disease, n (%)	6 (4.0)	7 (3.66)	0.880
Atrial fibrillation, n (%)	14 (9.3)	18 (9.42)	0.920
Smoking, n (%)	53 (35.3)	12 (6.20)	< 0.001
Diabetes mellitus, n (%)	28 (18.6)	55 (28.79)	0.030
Alcohol consumption, n (%)	3 (2.0)	4 (2.10)	0.950

* Data are presented as mean ± SD. SD: Standard deviation; TIA: Transient ischemic attack

**Figure 2:**
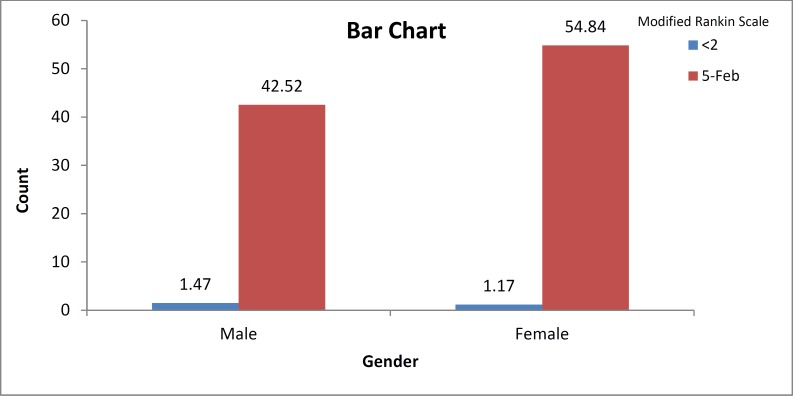
Modified Rankin Scale in both genders

Figure 2 depicts mRS ranking in both genders. No significant difference was found between two genders (P = 0.510). Mortality rate was 7.0% (4.7% in male and 8.9% in female). In spite of the high mortality rate in female, the difference was non-significant (P = 0.140). AF patients showed a higher mortality rate [18.8% (6 cases)] than non-AF patients [5.8% (18 cases)] (P = 0.010). Significant variables between two genders, that is, hypertension, diabetes, and smoking were assessed using logistic regression analysis; and as a result, hypertension [P = 0.03, odds ratio (OR) = 1.72, confidence interval (CI) = 1.04-2.84] and smoking (P < 0.001, OR = 2.18, CI = 1.81-3.56) were better predictor of stroke in males, compared with females. Diabetes failed to show a significant role in this model (P = 0.110). Smoking exerted high harmful effects in causing stroke, compared with hypertension.

## Discussion

In the present study, the mean age of the patients was 68.94 ± 12.74 years. Males and females accounted for 44% and 56% of the stroke patients. Barrett et al.^20^ and Martinez-Sanchez et al.^21^ found a higher number of males stroke patients in their studies; however, Salihovic et al. showed a higher number of female stroke patients (52.3%).^22^ Borhani-Haghighi et al. in a study from southern Iran found males had a higher number of stroke than females.^8^ In the present study, females were 2.3 years older than males. This difference was lower than that found in the other previously published studies.^6^^,^^9^^,^^23^^-^^26^ Bhattacharjee et al. showed that there was no significant difference in the mean age of male and female stroke patients.^27^ Our findings demonstrated that hypertension, smoking, and diabetes as the most prevalent risk factors showing a significant difference between two genders. The results of the present study were not in agreement with those reporting a higher frequency of AF in females.^10^^,^^21^^,^^23^^,^^24^ Moreover, Bhattacharjee et al. showed an almost equal AF rhythm in female and male stroke patients.^27^ Our results corroborated the findings of Salihovic et al. in which hypertension and diabetes were found at a higher rate in female stroke patients, as was smoking in male stroke patients.^22^ By contrast, Martinez-Sanchez et al. reported that hypertension, hyperlipidemia, drinking were present at higher rate in males.^21^ Bhattacharjee et al. found that the rate of hypertension, diabetes, ischemic cardiac diseases, and dyslipidemia is equal in two genders undergoing stroke, despite a higher rate of smoking and previous stroke in male stroke patients.^27^ Interestingly, there was a higher frequency of smoking in male and higher frequency of hypertension in female stroke patients in most reports. In reviewing the studies on stroke risk factor from Iran, we see: in Khorasan, the most common cause of posterior circulation stroke was atherosclerosis and rheumatic mitral stenosis; in Babol and Qom: the most common risk factor of stroke were hypertension and diabetes mellitus.^28^^-^^30^

In the present study, mRS in female was 0.17 times as severe as that in male, although this difference was not significant. Some studies showed more severe strokes based on mRS in females,^10^^,^^21^^,^^24^ despite some reports showing a higher mRS in male stroke patients.^27^ We also found a mortality rate of 7.0% that was marginally higher than that found by Heuschmann et al. (4.9%).^31^ Some reports, however, show that during the initial 30 days, the mortality rate after hospitalization due to stroke was higher in females.^21^^,^^24^^,^^32^

## Conclusion

The evidence from this study indicates that despite the differences in the risk factors of stroke between two genders, the severity of ischemic stroke and its outcome are equal in both genders. Planning a long follow-up and having larger sample size and multicenter studies in Iran would help us to better understand the role stroke risk factors in there.

## Notes

**How to cite this article: **Talebi M, Ghertasi M, Taheraghdam A, Andalib S, Sharifipour E. A comparison of risk factors and severity of ischemic stroke in female and male genders in North-West Iran: A cross-sectional study. Iran J Neurol 2014; 13(4): 215-9.
